# Unilateral Muscle Artifacts due to Non-compliance During Uptake Phase of ^18^F-FDG PET/CT in an Oncologic Patient

**DOI:** 10.4274/mirt.02419

**Published:** 2018-02-01

**Authors:** William Makis, Emmanuel W. Hudson

**Affiliations:** 1 Cross Cancer Institute, Department of Diagnostic Imaging, Edmonton, Canada

**Keywords:** Non-compliance, artifact, pitfall, muscle, musculoskeletal, ^18^F-FDG, PET

## Abstract

A 49-year-old male patient with a prior history of poor compliance with medical appointments was referred for an ^18^F-fluoro-2-deoxy-D-glucose (^18^F-FDG) positron emission tomography/computed tomography (PET/CT) for the staging of a rectal squamous cell carcinoma. The PET/CT showed unilateral diffuse skeletal muscle ^18^F-FDG uptake as well as bilateral salivary gland uptake artifacts, suggestive of non-compliance with patient preparation instructions. The PET/CT nurse noted that during the ^18^F-FDG uptake phase, the patient appeared intoxicated, and she found two beer cans hidden in the waste disposal beside his chair just prior to imaging. The patient only admitted to eating a cookie approximately 30 minutes after the injection of ^18^F-FDG PET/CT and denied consuming alcohol during the uptake phase. We present the imaging findings of non-compliance with patient instructions during the uptake phase of ^18^F-FDG.

## Abstract (tr)

Kırk dokuz yaşında doktor randevularına uyumsuz olduğu bilinen bir erkek hasta rektal skuamöz hücreli karsinom evrelemesi amacıyla ^18^F-fluoro-2-deoksi-D-glukoz (^18^F-FDG) pozitron emisyon tomografisi/bilgisayarlı tomografi (PET/BT) için yönlendirilmişti. PET/BT’de unilateral diffüz iskelet kasında ^18^F-FDG tutulumu, bilateral tükrük bezi artefaktları görüldü, bulguları hastanın hazırlık talimatlarına uyumsuzluğu olarak değerlendirildi. PET/BT hemşiresi ^18^F-FDG tutulumu sırasında hastanın intoksike görünümde olduğunu farketti ve görüntülemeden önce hastanın iskemlesinin yanındaki çöp kutusunda iki bira şişesi buldu. Hasta ^18^F-FDG PET/BT enjeksiyonundan 30 dakika sonra bir kurabiye yediğini ancak alkol almadığını belirtti. Burada hazırlık talimatlarına uyumsuz bir hastada ^18^F-FDG tutulum fazındaki görüntüleme bulgularını sunuyoruz.

## Figures and Tables

**Figure 1:**
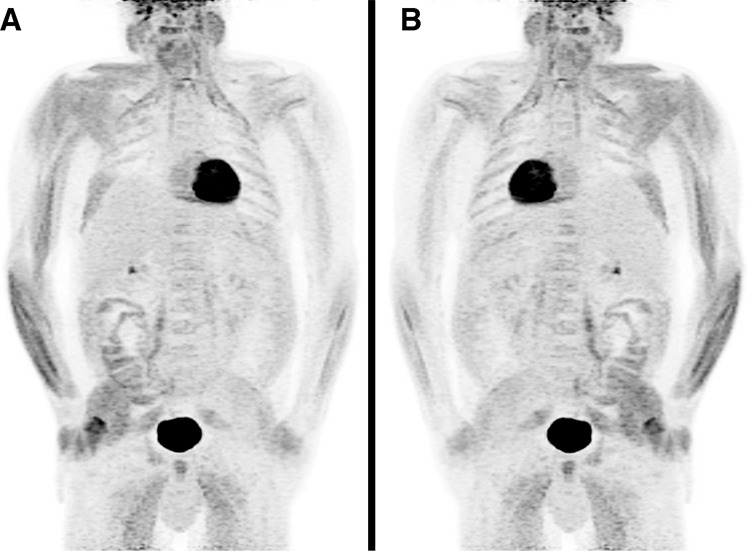
A 49-year-old male patient had ^18^F-fluoro-2-deoxy-D-glucose (^18^F-FDG) positron emission tomography/computed tomography (PET/CT) (Biograph-mCT, Siemens, Germany) to stage a rectal squamous cell carcinoma. He was injected with 352 MBq of ^18^F-FDG and his blood glucose was 6.8 mmol/L just prior to injection. During the 60-minute uptake phase prior to imaging, the nurse observed the patient: he was sitting and leaning on one side, appeared intoxicated and his breath smelled of alcohol. He was constantly moving in his seat. Two beer cans were found in the waste disposal next to his chair just prior to imaging. On questioning, he denied drinking alcohol and only admitted to eating a cookie at approximately 30 minutes after ^18^F-FDG injection. PET/CT maximum intensity projection images with (A) anterior and (B) posterior views revealed diffuse intense unilateral muscle ^18^F-FDG uptake artifacts involving the right shoulder, arm, hand, right chest wall and right gluteus muscles.

**Figure 2:**
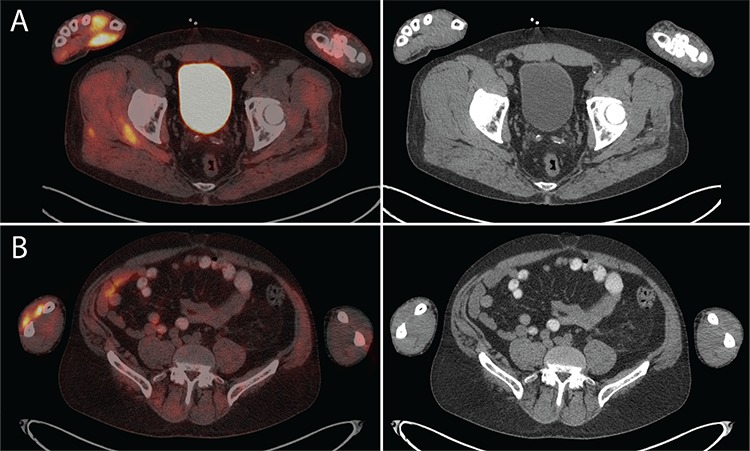
(A) Intense ^18^F-FDG uptake in the right thenar eminence with maximum standard uptake value (SUV_max_) 8.8 was the most intensely ^18^F-FDG avid abnormality in the entire PET/CT study, and was most likely the result of the patient holding beer cans and drinking beer. (B) Right dorsal extensor forearm muscles also showed intense ^18^F-FDG uptake with SUV_max_ 6.7.

**Figure 3:**
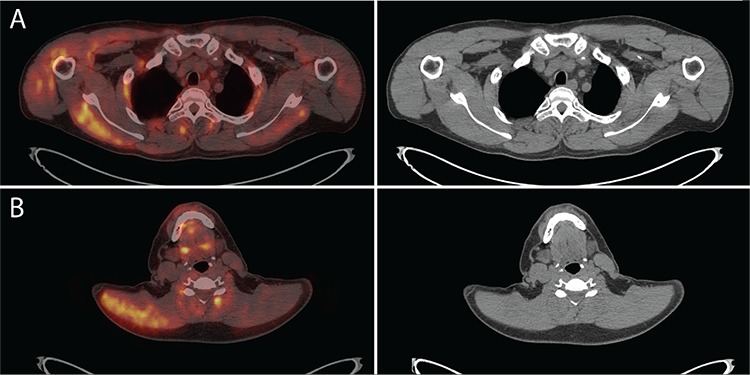
Right biceps muscles showed increased ^18^F-FDG uptake with SUV_max_ 5.0, (A) right shoulder muscles had SUV_max_ 4.0, (B) right trapezius muscle had SUV_max_ 4.1 and right serratus anterior muscle had SUV_max_ 3.8.

**Figure 4:**
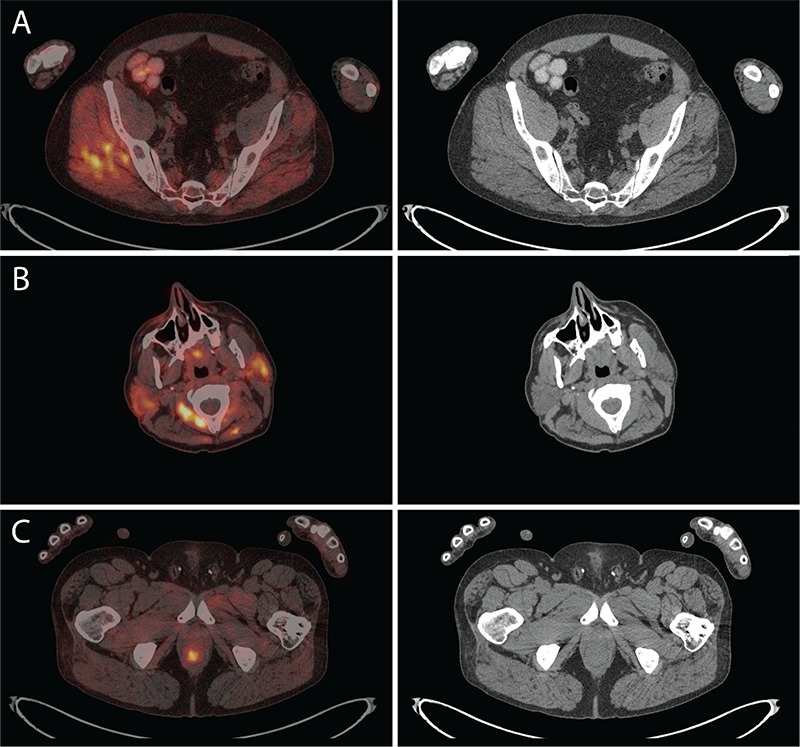
(A) Right gluteal muscles showed increased ^18^F-FDG uptake with SUV_max_ 5.3, and (B) both parotid glands showed increased ^18^F-FDG uptake with SUV_max_ 4.7 in the right and SUV_max_ 4.8 in the left, most likely due to alcohol/food consumption. (C) The rectal primary lesion had intense ^18^F-FDG uptake with SUV_max_ 5.2 and there was no evidence of distant metastases. The ^18^F-FDG physiologic uptake artifacts from physical activity and movements of the right hand, arm, shoulder and gluteus maximus (as well as drinking and eating), with normal corresponding CT findings, did not interfere with cancer staging. It is important that a patient is relaxed at time of ^18^F-FDG injection and has avoided vigorous exercise in the hours leading up to the PET/CT. Most authors recommend avoiding physical exercise 24 hours before ^18^F-FDG administration (1,2,3,4), although vigorous exercise up to 4 days before imaging has been reported to cause muscle ^18^F-FDG uptake artifacts (5). Diffusely increased ^18^F-FDG uptake in muscles, as a PET/CT artifact, has been described in the following situations: insulin administration (6), voluntary physical activity such as chewing gum, exercise or sexual activity (4,5,7), involuntary physical activity such as labored breathing or muscle spasms (8), post surgical changes (9,10), post radiation inflammation (11), dermatomyositis (12), infection (10,13,14), or post injection (15). Unilateral intense muscle ^18^F-FDG uptake is rare and has been described in a few cases such as hemiparesis from stroke (16), in patients with multiple sclerosis (17), due to tracer injections (15) or more commonly in certain head and neck muscles (18). Non-compliance with patient instructions during the uptake phase of ^18^F-FDG can cause significant artifacts and recent examples in the literature include smartphone use (19), reading a small book and even tapping the foot (20).
